# Beyond the Genome: Deciphering the Role of MALAT1 in Breast Cancer Progression

**DOI:** 10.2174/0113892029305656240503045154

**Published:** 2024-05-22

**Authors:** Md Sadique Hussain, Mohit Agrawal, Nusratbanu K. Shaikh, Nikita Saraswat, Gurusha Bahl, Mudasir Maqbool Bhat, Navneet Khurana, Ajay Singh Bisht, Muhammad Tufail, Rajesh Kumar

**Affiliations:** 1 School of Pharmaceutical Sciences, Jaipur National University, Jaipur, Rajasthan (302017), India;; 2 Department of Pharmacology, School of Medical & Allied Sciences, K.R. Mangalam University, Gurugram 122103, India;; 3 Department of Quality Assurance, Smt. N. M. Padalia Pharmacy College, Ahmedabad, 382210, Gujarat, India;; 4 Department of Pharmaceutical Sciences, University of Kashmir, Srinagar, Jammu and Kashmir, India;; 5 School of Pharmaceutical Sciences, Lovely Professional University, Phagwara, Punjab, India;; 6 School of Pharmaceutical Sciences, Shri Guru Ram Rai University, Patel Nagar, Dehradun, Uttarakhand (248001), India;; 7 Department of Oral and Maxillofacial Surgery, Center of Stomatology, Xiangya Hospital, Central South University, Changsha, China

**Keywords:** Breast cancer, MALAT1, lncRNAs, molecular interactions, therapeutic implications, quality of life

## Abstract

The MALAT1, a huge non-coding RNA, recently came to light as a multifaceted regulator in the intricate landscape of breast cancer (BC) progression. This review explores the multifaceted functions and molecular interactions of MALAT1, shedding light on its profound implications for understanding BC pathogenesis and advancing therapeutic strategies. The article commences by acknowledging the global impact of BC and the pressing need for insights into its molecular underpinnings. It is stated that the core lncRNA MALAT1 has a range of roles in both healthy and diseased cell functions. The core of this review unravels MALAT1's multifaceted role in BC progression, elucidating its participation in critical processes like resistance, invasion, relocation, and proliferating cells to therapy. It explores the intricate mechanisms through which MALAT1 modulates gene expression, interacts with other molecules, and influences signalling pathways. Furthermore, the paper emphasizes MALAT1's clinical significance as a possible prognostic and diagnostic biomarker. Concluding on a forward-looking note, the review highlights the broader implications of MALAT1 in BC biology, such as its connections to therapy resistance and metastasis. It underscores the significance of deeper investigations into these intricate molecular interactions to pave the way for precision medicine approaches. This review highlights the pivotal role of MALAT1 in BC progression by deciphering its multifaceted functions beyond the genome, offering profound insights into its implications for disease understanding and the potential for targeted therapeutic interventions.

## 1 INTRODUCTION

Breast cancer (BC) represents a significant global health challenge. As reported by the American Cancer Society, with an anticipated 2.3 million new cases in 2020, it is the most common cancer detected in women. Despite a 40% reduction in the BC death rate between 1989 and 2017, it still ranks overall as the biggest cause of fatalities due to cancer for women globally. Projections for the global cancer burden indicate an anticipated increase to 28.4 million cases by 2040, driven primarily by demographic shifts and escalating risk factors associated with lifestyle changes, particularly evident in transitioning countries [1-3]. These risk factors span genetic and non-genetic elements, encompassing age, gender, family history, reproductive factors, and lifestyle determinants, such as alcohol consumption, physical inactivity, and obesity [4]. The early detection of BC is paramount for effective management. Current diagnostic modalities encompass diagnostic imaging and biopsy. Circulating microRNAs (miRNAs) are one type of non-invasive biomarker that has become an intriguing possibility for diagnostics. Their clinical applicability remains a subject of ongoing investigation [5, 6].

Among the several types of non-coding RNAs, long non-coding RNAs (lncRNAs) are distinguished by their length, usually being more than 200 nucleotides. These molecules exhibit remarkable spatiotemporal specificity and find themselves involved with a wide spectrum of physiological mechanisms and disease states [7, 8]. One of the pivotal roles ascribed to lncRNAs is their involvement in the control of apoptosis by altering signalling pathways linked with autophagy, which notably include the PI3K/AKT/mTOR signalling pathway. Consequently, these lncRNA-mediated mechanisms have profound implications in the progression of radio-resistance and chemoresistance in relation to carcinoma [9, 10]. Within the nervous system, lncRNAs manifest varying degrees of expression following neural growth, development, injury, and degeneration, thereby exerting influence over diverse physiological processes in the nervous system [11]. Furthermore, emerging research underscores the differential expression of lncRNAs across a spectrum of diseases, encompassing conditions like bladder cancer, psoriasis, and systemic lupus erythematosus. This differential expression has resulted in the recognition of lncRNAs as possible non-invasive biomarkers for diagnostic purposes [12-16]. Table **1** lists the functions of some of the lncRNAs in carcinoma of the breast.

Metastasis-Associated Lung Adenocarcinoma Transcript 1 (MALAT1) represents a lncRNA that exhibits notable overexpression across many cancer forms, such as gastric cancer (GC), esophageal cancer (EC), and non-small cell lung cancer (NSCLC) [17, 18]. The multifaceted role of MALAT1 extends to the orchestration of fundamental biological functions, encompassing gene transcription, cell division, invasion, autophagy, migration, and apoptosis [17, 19, 20]. This lncRNA acts like competitive endogenous RNA denoted as ceRNA, effectively sequestering miRNAs and modifying targeted gene function [17, 19, 21]. Furthermore, MALAT1 is essential for controlling carcinogenic signalling. pathways, including but not limited to TGF/SMAD, Hippo, Wnt/β-catenin pathways, and JAK/STAT [17, 22]. Significantly, MALAT1 shows effectiveness as a biomarker for multiple healthcare applications in NSCLC management. It holds promise to discover diseases promptly as a diagnostic tool, an indicator of disease severity, and an element in prognosis evaluation for NSCLC patients [17].

Research was conducted on MALAT1's role in BC invasion and cell proliferation, its potential as a therapeutic target, its clinical applicability, and its prognostic significance. Our study aims to offer a comprehensive insight into the involvement of MALAT1 in BC advancement. The discoveries made will augment our comprehension of the molecular processes that underlie BC and may carry substantial implications for the innovation of innovative diagnostic, prognostic, and therapeutic approaches for this debilitating ailment. Ultimately, the revelation of MALAT1's role in BC progression could lay the groundwork for more precise and efficacious interventions, resulting in enhanced patient outcomes and quality of life (QoL).

## 2 MALAT1: MOLECULAR STRUCTURE AND FUNCTION

### 2.1 Molecular Structure of MALAT1

In 1997, research on the molecular sense linked to multiple endocrine neoplasia (MEN) type 1 revealed the transcript of the MALAT1 gene to be “alpha” transcripts [46, 47]. Subsequently, in 2003, MALAT1 connected to metastasis in early-stage NSCLC patients [48]. Despite its prominent role in cancer, MALAT1 remains not solely found in cancerous cells and exhibits widespread expression across various tissues and cell types. Interestingly, the MALAT1 sequence is conserved across a total of '33 mammalian species, and putative substitutes with moderate sequence similarity are found on syntenic chromosomes in lower vertebrates [49-51]. MALAT1 was identified in 2007 as a prevalent nuclear transcript that was mostly found in nuclear speckles, which are domains that are known to be involved in pre-mRNA splicing and storage [50]. The MALAT1 RNA is produced *via* the tRNA generation route and noticeably lacks a poly(A) tail at its 3' end [51]. The mature MALAT1 transcripts and the smaller amount of MALAT1-associated small cytoplasmic RNA (mascRNA) are produced when the original transcript, which has a tRNA-like structure at its 3' terminus, is cleaved by tRNA reprocessing endonucleases, such as RNase P and RNase Z. Additionally, MALAT1 RNA's 3' end takes on a unique triple-helix shape that is similar to that of the viral RNA of Kaposi's sarcoma-associated herpesvirus (KSHV), which is known as its gene expression and nucleus persistence component [52, 53]. This framework consists of three helixes that confer resistance to nucleases and enhance translational efficiency upon cytoplasmic export. Recently, the crystal framework of triple helixes was elucidated, offering detailed insights into the functional roles of individual nucleotides in preserving the unusual triple-helix configuration [52, 54]. It is noteworthy that sequence conservation amongst mammalian MALAT1 and zebrafish is primarily observed in the 3' end, highlighting the significance of preserved 3' end processing [51]. MALAT1's half-life in human tissues is 9–12 hours, which is longer compared to other common ncRNAs. This may be because MALAT1 has a triple-helix configuration at the 3' end of its molecule [55-57].

### 2.2 Functions of MALAT1

MALAT1 exerts its influence on gene expression through diverse mechanisms. For instance, in one study, by means of the mechanism of miR-23b-3p, MALAT1 has been shown to promote α-synuclein production, which in turn causes autophagic impairment, an inflammatory response in microglia and eventually mortality in dopaminergic neuronal cells [58]. An additional study revealed that the MALAT1-KTN1-EGFR regulatory axis exists and has a role in the development of cutaneous squamous cell carcinoma [59]. Hypoxia in BC cells has been shown to trigger chromatin interactions unique to cancer cells, which raises MALAT1 transcription [60]. Additionally, MALAT1 serves as a miR-26a-5p sponge, modulating Smad1 and thus influencing colorectal cancer progression by orchestrating autophagy [61]. Additionally, MALAT1 has been associated with promoting aggressive breast cancer (BC) by inducing overexpression of MALAT1 and simultaneous downregulation of hsa-miR-448 [62]. In a different context, MALAT1 has also been identified as a contributor to the development of retinal damage caused by diabetes *via* miR-378a-3p to trigger PDE6G [63]. These collective findings underscore the versatile role of MALAT1 in orchestrating gene expression across various cancer types, each characterized by distinct molecular mechanisms.

### 2.3 Previous Research Linking MALAT1 to Cancer Development

Extensive research has established a compelling connection between MALAT1 and the onset of cancer. Overexpression of MALAT1 has been consistently observed across various cancer types, encompassing colorectal cancer, prostate cancer, pancreatic cancer, and BC, among others (Table **2**) [64-69]. In the realm of cancer biology, MALAT1 emerges as a multifaceted regulator, influencing gene expression, cell proliferation, migration, invasion, and autophagy through an array of mechanisms, notably including miRNA sequestration and the modulation of target gene expression [64-66, 68, 70]. Furthermore, MALAT1 exerts its impact on cancer progression by intricately interacting with several oncogenic signalling pathways, including but not limited to the TGF/SMAD, Hippo, JAK/STAT, and Wnt/β-catenin pathways [65, 67]. Apart from its legislative function, MALAT1 exhibits promise as a biomarker for many therapeutic uses, encompassing early cancer diagnosis, assessment of disease severity, and prognosis evaluation [65, 68]. Collectively, these research findings underscore the substantial involvement of MALAT1 in the complex terrain of cancer evolution and progression, highlighting the need for further investigations to elucidate its specific roles within distinct cancer types.

## 3 MALAT1'S IMPACT ON BREAST CANCER PROGRESSION

MALAT1 has emerged as a key player in the development of BC. Numerous studies have thoroughly examined its impact on several facets of BC biological science, such as drug resistance, invasion, migration, and expansion of cells [90-93]. Shao *et al.* reported that MALAT1 contributes significantly to the advancement of BC as a sponge for miR-101-3p, *via* promoting mTOR/PKM2 signalling [94]. In clinical BC specimens, another investigation revealed an inverse relationship between MALAT1 expression and miR-143-3p scales. Notably, miR-143-3p was found to be downregulated in pathological tumour cases when contrasted with healthy subjects. In the quest to identify MALAT1's target miRNAs, computational predictions were made using the Starbase 2.0 repository. Among potential candidates, miR-101-3p emerged as a promising mark. Experimental validation of this interaction involved the introduction of miR-101-3p mimics, which resulted in decreased luciferase activity in BC cells transfected with MALAT1-WT. Nevertheless, this impact was not seen in BC cells injected with MALAT1-Mut, where the binding site had been modified. Moreover, the impact of siRNA-mediated MALAT1 knockdown on miR-101-3p levels was explored, revealing a significant increase in miR-101-3p levels in BC cells transfected with si-MALAT1 in comparison to those transfected with si-NC. Significantly, Spearman's correlation study showed that the levels of miR-101-3p and MALAT1 were inversely correlated [94]. In another investigation, a different relationship between levels of miR-143-3p and MALAT1 expression in clinical BC samples was found. Interestingly, in comparison with healthy individuals, miR-143-3p was shown to be diminished in the symptomatic tumour tissues [95].

**Fig. (1):**
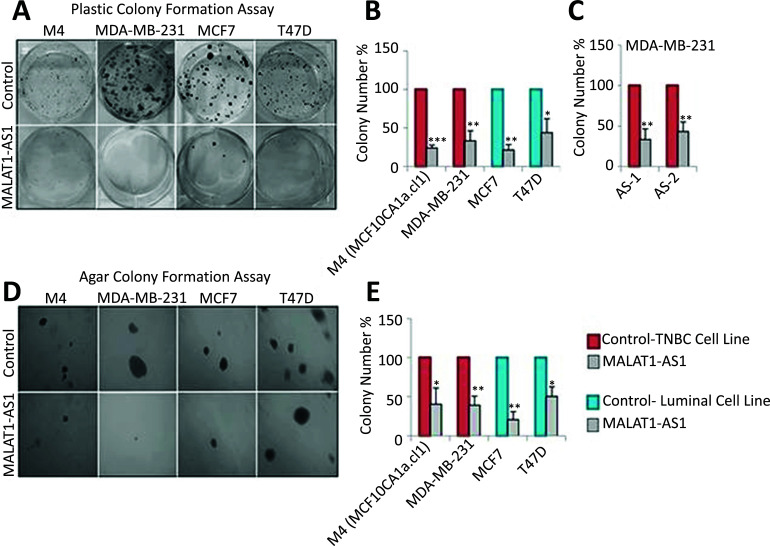
Reducing the expression of MALAT1 in breast cancer cells results in a decrease in cell proliferation and the formation of colonies without anchorage. (**A** and **B**). Cell proliferation is decreased in all subtypes of breast cancer when MALAT1 is depleted with DNA antisense oligonucleotides. Clonogenic tests are used to measure cell proliferation (plastic colony formation). (**C**). MDA-MB-231 cells treated with two different MALAT1-specific antisense oligonucleotides or control oligonucleotides are used in the plastic colony formation experiment. (**D** and **E**). In various cell lines, anchorage-independent growth is reduced when MALAT1 is depleted. Three distinct biological repeats produced the data shown in A–E, with error bars denoting the standard error of the mean (SEM) [99].

Furthermore, a separate study showed that the suppression of MALAT1 enhanced cell apoptosis and sensitized BC cells to taxanes and adriamycin, bolstering their responsiveness to these drugs [96]. Complementing these primary research findings, a comprehensive review article delved into MALAT1's structural and functional aspects, examining its expression patterns across several BC categories [97]. The complex relationships across MALAT1 and miRNAs were also examined in this review, which helped to clarify the various signalling networks connected to BC [90]. Finally, a study demonstrated how important hypoxia-induced MALAT1 is for BC cells to migrate and grow through the sequestration of miR-3064-5p [98]. Collectively, these studies underscore MALAT1's pivotal role in BC progression, hinting at its capability as a therapy goal in the framework of treating BC. Jadaliha *et al*. conducted a study to explore MALAT1's function in tumour growth. They achieved this by reducing MALAT1 levels in BC lineages of cells representing many subclasses. The researchers assessed the impact of this depletion on various cancer-related characteristics, including anchorage-dependent growth and cell proliferation, as determined through gene-editing assays using plastic plates and development that is anchorage-independent using flexible agar colonies tests Since epithelial cells exhibit the highest level of MALAT1 expression, the study first used modified antisense oligonucleotides (ASOs) to target lines of luminal cells, namely T47D and MCF7 cells, as shown in Fig. (**1**). The impact of MALAT1 depletion on the perpetual growth of colonies in polyethylene plates that are dependent on anchoring was examined by the researchers. Notably, decreased proliferation cells were the outcome of MALAT1 reduction in luminal cells (Fig. **1**) [99].

**Fig. (2):**
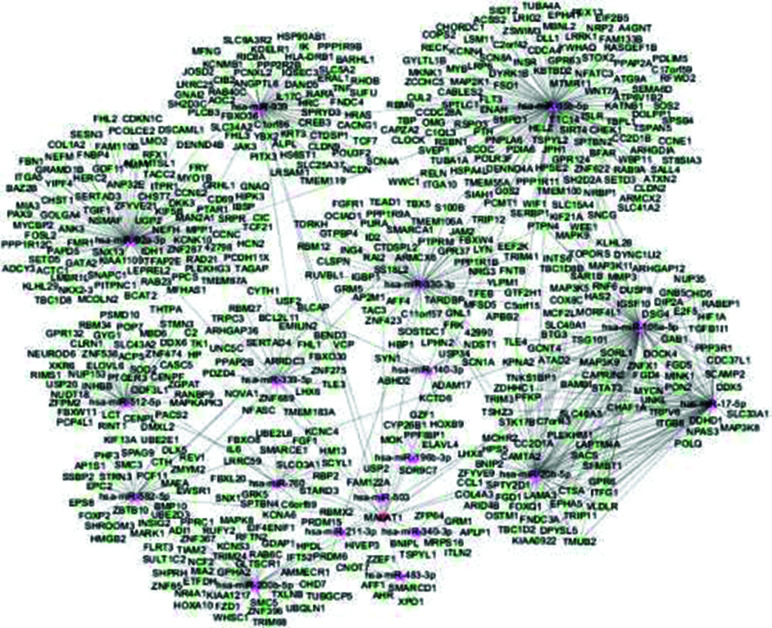
Network representation of the interactions among MALAT1, miRNAs, and mRNA targets in BC. This visual representation illustrates the complex regulatory relationships between these molecular entities and provides insights into their potential roles in BC pathogenesis [100].

To investigate whether MALAT1 operates through a similar mechanism, Zheng *et al.* conducted a bioinformatics investigation to identify miRNAs that might have an interaction with MALAT1 and their target genes. They identified 18 miRNAs associated with BC that might have regulatory roles on MALAT1. To enhance the reliability of target prediction, they imposed specific criteria: a maximum folding free energy of ≤25 and a system score of >160. To reduce the risk of false positives, our criteria for considering miRNA-mRNA pairs required confirmation by a minimum of two distinct prediction applications. This stringent approach led to identifying 24 genes predicted to be targets of dysregulated miRNAs. Subsequently, they constructed a MALAT1-miRNA-mRNA regulatory network to visualize these interactions (Fig. **2**) [100].

## 4 MECHANISMS OF ACTION: HOW MALAT1 DRIVES BREAST CANCER PROGRESSION

### 4.1 MALAT1 as a Modulator of Cell Proliferation and Survival

MALAT1 has demonstrated involvement in promoting cell proliferation and survival across diverse cancer types. Among them are osteosarcoma, esophageal squamous cell carcinoma (ESCC), and ovarian cancer [101, 102]. In ovarian cancer, MALAT1 is often upregulated and has been associated with lower individual survival rates, indicating reduced life expectancy and increased risk of disease progression. Targeting MALAT1 through siRNA knockdown significantly diminishes ovarian cancer cell viability, impedes migration, and reduces invasion capabilities [103]. Similarly, in osteosarcoma, MALAT1 is overexpressed and serves as a predictor of unfavourable patient survival. E-cadherin expression rises and osteosarcoma cell invasion is reduced when MALAT1 is suppressed. In terms of its mechanism of action, the transcriptional upregulation of MALAT1 is stimulated by TGF-β and exhibits a strong correlation with EZH2. The expression of E-cadherin is suppressed in part by this interaction [104]. MALAT1 functions as an apoptotic inhibitor in the context of esophageal squamous cell carcinoma (ESCC), thereby inhibiting miR-590-3p and boosting migration, cell proliferation, epithelial-mesenchymal transition (EMT), and invasion [105]. In a variety of cancer types, MALAT1 is shown to play a key role in promoting cell survival as well as growth.

**Fig. (3):**
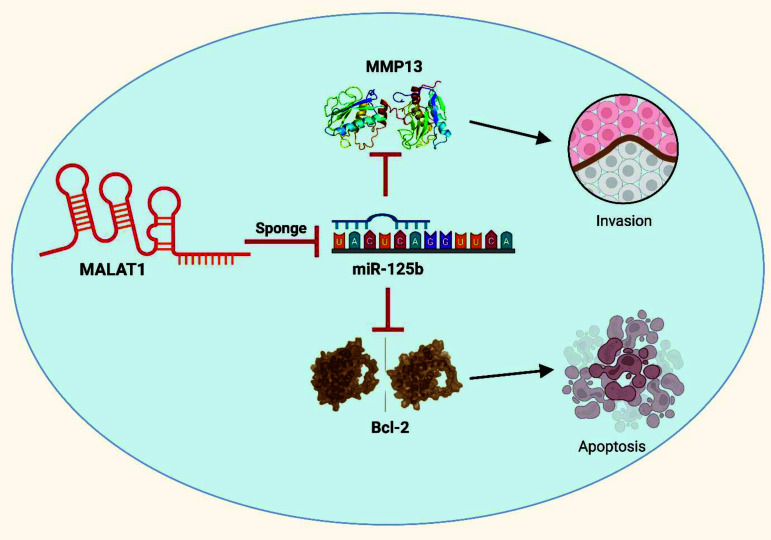
The graphic representation illustrates the interconnection between MALAT1, miR-125b, Bcl-2, MMP13 and the processes of apoptosis or invasion [109].

Several investigations have examined MALAT1's role in controlling BC cell survival and proliferation. Specifically, one investigation revealed that inhibiting MALAT1 expression caused a discernible decline in the proliferation of BC cells, accompanied by decreased invasion and migration capabilities. Furthermore, this intervention caused cell cycle interruption at the G1 phase and encouraged apoptosis [106]. Another study focusing on MDA-MB-361 BC cells, reported that deletion of MALAT1 increased apoptosis while simultaneously diminishing cell proliferation. Additionally, a separate study demonstrated that metformin treatment led to an upregulation in MALAT1 expression within BC cells [107]. Importantly, it was observed that the combined approach of metformin treatment and MALAT1 knockdown enhanced the anti-proliferative effects of metformin [108]. Xie *et al.* indicated that MALAT1 may promote cancer development and invasion by reducing the levels of matured miR-125b and increasing the expression of certain genes, such as Bcl-2 and MMP-13 (Fig. **3**). The circuit known as “MALAT1-miR-125b-Bcl-2/MMP-13” has been associated with the progression of BC, suggesting a potential therapeutic approach for this disease [109]. All the above evidence points to MALAT1's important control over BC cellular survival and expansion, and they also point to the protein's viability as an avenue for BC therapy.

### 4.2 MALAT1's Involvement in EMT

MALAT1 has emerged as a key contributor to the induction of EMT across diverse cancer types. In the context of colorectal cancer, YAP1-induced MALAT1 is currently recognised as an effective motivator of EMT and angiogenesis, facilitated through its sponge-like properties for miR-126-5p [110]. Similarly, in the event of prostate cancer, MALAT1 has been associated with the promotion of EMT while simultaneously inhibiting apoptosis. Notably, treatment with quercetin, leading to MALAT1 downregulation, has demonstrated a capacity to counteract EMT induction and promote apoptosis [111]. In EC, MALAT1 has been found to exert its influence on EMT through the Ezh2-Notch1 signalling pathway [112]. Additionally, in lung cancer, MALAT1 has been implicated in inducing EMT and fostering brain metastasis [113]. Cumulatively, MALAT1 appears to assume a pivotal role in driving EMT processes across various cancer types.

Numerous investigations were conducted into the contribution of MALAT1 in the process of EMT in BC. Specifically, one study elucidated that MALAT1 promotes EMT by means of the MALAT1/miR-26b/HMGA2 orientation [114]. Additionally, another investigation unveiled the role of the ribonucleic complex HuR-MALAT1 in repressing CD133 expression and, consequently, inhibiting EMT in BC [89]. Furthermore, a third study demonstrated the significance of the miR-204/ZEB2 tilt as a pivotal moderator in MALAT1-induced EMT within BC [115]. Moreover, an independent study highlighted how inhibition of MALAT1 triggers EMT through the PI3K-AKT cascade in BC [116]. Lastly, another study emphasized the crucial MALAT1's role in activating EMT in BC instances thriving in an acidic microenvironment [117]. In summary, these collective findings underscore the substantial role of MALAT1 in orchestrating EMT in breast cancer and imply that it might be used as an approach to reduce the spread of BC.

### 4.3 MALAT1-mediated Angiogenesis and Dissemination

Research Assessments have shown MALAT1's possible role in processes related to angiogenesis and metastasis. For instance, in the context of myocardial infarction (MI), a study revealed that M1-BMMs-EVs played a role in inhibiting angiogenesis and MI post-MI by modulating the MALAT1/miR-25-3p/CDC42 direction [118]. In the domain of BC, investigations have demonstrated that MALAT1, when delivered *via* BC cell-derived Exosomes (Exo), can augment the chemoresistance of BC cells and the malignant characteristics [119]. Conversely, certain studies have suggested a therapeutic potential in targeting MALAT1 to counteract angiogenesis and metastasis. In prostate cancer, miR-423-5p was identified as an inhibitor of MALAT1-mediated proliferation and metastasis [67]. Similarly, in OSCC, silencing METTL14, which induced m6A modification of MALAT1, emerged as a therapeutic strategy with implications for treating OSCC by means of the MALAT1/miR-224-5p/KDM2A core [120].

Emerging evidence indicates that MALAT1 may contribute to angiogenesis and metastasis in BC. In one study, it was revealed that JAG1 promotes angiogenesis in triple-negative BC by enhancing Exo secretion, thereby activating MALAT1-miR-140-5p-JAG1/VEGFA tangent [121]. Another research effort showed that MALAT1-containing BC cell-derived exo may improve the aggressive traits and chemical resistance of BC cells [119]. Furthermore, MALAT1 was implicated in the promotion of angiogenesis in BC through the regulation of VEGF expression [122]. Collectively, these outcomes suggested an essential aspect for MALAT1 in fostering angiogenesis and metastasis in BC, underscoring the need for further research to unravel its intricate mechanisms and explore potential therapeutic applications.

### 4.4 Interactions with Key Oncogenic and Tumor Suppressor Pathways

Limited information is available concerning the interactions between MALAT1 and important tumour suppressant and carcinogenic networks in the backdrop of BC. Nevertheless, a study has observed that MALAT1 plays a part in BC evolution, assuming as a miR101-3p exfoliator, thereby facilitating mTOR/PKM2 signalling [94]. In a separate investigation focused on BC, there was a negative relationship between the levels of EGFR and SHP-1 expression [123]. Although these studies do not directly elucidate MALAT1's interactions with key oncogenic and tumor suppressor pathways in BC, they provide insightful information about the fundamental molecular processes driving the advancement of BC. To obtain a thorough comprehension of MALAT1's function in BC and its interactions with these pathways, further research is warranted.

## 5 CLINICAL IMPLICATIONS AND BIOMARKER POTENTIAL

### 5.1 MALAT1 as an Ideal Predictor for Prognosis in Breast Cancer Research

MALAT1 has emerged as a candidate prognostic biomarker in BC. In a study, notable upregulation of MALAT1 showed promise in distinguishing high-risk subjects from healthy subjects, while concurrent downregulation in GAS5 levels was associated with metastasis and recurrence, further highlighting their prognostic significance [124]. Furthermore, MALAT1 has garnered substantial attention in the context of BC, with multiple studies exploring its implications [90]. However, it is vital to acknowledge that BC prognosis may also be influenced by a plethora of other biomarkers, including tumor-infiltrating B cells, progranulin and sortilin, and ATOX1, each offering unique insights into prognosis prediction [125-127]. Consequently, further extensive research is imperative to discern the most efficacious biomarker or combination thereof for accurate prognosis prediction in BC.

### 5.2 Challenges and Considerations in Targeting lncRNAs for Therapy

LncRNAs have gained recognition as essential modulators of gene expression across diverse diseases, prominently within the realm of cancer. The dysregulation of lncRNAs has been conspicuously observed across a spectrum of tumor types, propelling them into the spotlight as prospective diagnostic, prognostic, and therapeutic instruments [128, 129]. Yet, leveraging lncRNAs for clinical therapeutic interventions is hampered by a range of substantial challenges. Foremost among these challenges is the intricate and often elusive mechanisms of action governing lncRNAs, which pose a significant barrier to the creation of accurate and successful therapeutic approaches [130, 131]. Moreover, lncRNAs frequently exhibit low expression levels and tissue-specific patterns, confounding therapeutic endeavors by making selective targeting while sparing normal cells a formidable task [129]. Additionally, the medicinal compounds' release to specific sites is notably complicated, particularly in the context of disorders affecting the brain, where the formidable blood-brain barrier obstructs efficient drug delivery [132].

Notwithstanding these substantial challenges, recent investigations have yielded promising findings regarding the possibility of using lncRNA tailoring as a cancer treatment [129, 133]. However, it is essential to emphasize that thorough and diligent research efforts are imperative to overcome these challenges, enhance our understanding of lncRNA mechanisms, and ultimately facilitate the creation of effective and safe therapeutic approaches.

## 6 CHALLENGES AND FUTURE DIRECTIONS

MALAT1's contribution to medication susceptibility throughout BC remains an area with limited understanding and warrants further investigation. Preliminary research has indicated potential links between MALAT1 and drug resistance in BC. Specifically, one study unveiled that MALAT1 is implicated in promoting both BC progression and resistance to doxorubicin, with its mechanism of action involving the regulation of miR-570-3p [91]. Another study demonstrated the cell function with regards to hypoxia-induced MALAT1 promotes BC cell migration and proliferation through its interaction with miR-3064-5p [134]. While these findings offer tantalizing insights, it is imperative to acknowledge that a comprehensive comprehension of the intricate mechanisms and therapeutic potential of MALAT1 in BC drug resistance necessitates further rigorous investigation.

A sustained investigation into the engagement of MALAT1 in Breast Cancer is still imperative for the progression of our comprehension of this multifaceted ailment. Such endeavours are vital for the formulation of groundbreaking approaches to BC diagnosis, treatment, and patient management. By embracing and exploring these prospective avenues of research, we can continue to unravel the applicability of MALAT1 in the BC setting and potentially harness its utility as an asset within the realm of clinical oncology.

## CONCLUSION

BC is a significant global health concern, and its prevalence is expected to increase in the coming years. Early detection and effective treatment are essential for addressing this complex disease. Through our comprehensive research, we have uncovered the potential importance of MALAT1 in BC progression. Initially associated with metastasis in NSCLC, MALAT1 is widely expressed and possesses a unique molecular structure that contributes to its stability and functionality. MALAT1 exerts regulatory control over gene expression through various mechanisms, influencing critical mechanisms like invasion, migration, division, and autophagy. While MALAT1's involvement in multiple kinds of cancer, such as prostate and colon cancer, is well-documented, its roles and mechanisms can vary in different contexts. It interacts with multiple oncogenic signalling pathways, offering potential targets for therapeutic intervention. Additionally, MALAT1 holds value in BC as a predictive and therapeutic biomarker, potentially facilitating early detection and prognosis assessment. Its distinct expression patterns in various diseases highlight its promise as a biomarker for non-invasive diagnosis. However, further research is required to fully elucidate its specific functions in the context of BC, paving the way for innovative approaches to diagnosis, prognosis, and therapy. MALAT1 represents a promising avenue for future investigations and advancements in BC research and treatment. MALAT1 serves as a crucial guide in our exploration of ncRNAs, contributing to advancements in patient outcomes and quality of life for those affected by BC.

## AUTHORS’ CONTRIBUTIONS

It is hereby acknowledged that all authors take the responsibility for the manuscript's content and are consenting to its submission. They have meticulously reviewed all results and unanimously approved the final version of the manuscript.

## Figures and Tables

**Table 1:** Roles of different long non-coding RNAs in breast carcinoma.

**lncRNA**	**Gene Type**	**Functions**	**References**
ANCR	-	CI and CM	[23]
ARNILA	+	CI and CM	[24]
ATB	+	EMT	[25]
CHET1	+	CP, CI, and CM	[26]
EPIC1	+	Cell cycle progression	[27]
EZR-AS1	+	CP and CM	[28]
GACAT3	+	CP	[29]
ITGB2-AS1	+	CI and CM	[30]
LNC00511	+	CP and CI	[31]
Lnc015192	+	CI, CM, and EMT	[32]
MAGI2-AS3	-	CP	[33]
MEG3	-	CP and EMT	[34]
MIR100HG	+	CP	[35]
NEAT1	+	CM	[36]
NKILA	-	EMT	[37]
NNT-AS1	+	CP	[38]
P10247	+	CM	[39]
PDCD4-AS1	-	CP	[40]
PRLB	+	CP and CM	[41]
PTENP1	-	CP and CM	[42]
PVT1	+	CP	[43]
TUG1	+	CP and CM	[44]
UCA1	+	CM	[45]
XIST	-	CP	[66, 67]

**Abbreviations:** -, Suppressor; +, Oncogene; CI, Cellular invasion; CM, Cellular metastasis; CP, Cellular proliferation; EMT, epithelial-mesenchymal transition.

**Table 2:** Role of MALAT1 across different cancers.

**Type of Cancer**	**Function**			**References**
	**Apoptosis**	**Metastasis**	**Proliferation**	
Bladder	-	+	-	[71]
	+	+	+	[72]
Breast	-	+	+	[73]
Cervical	+	+	+	[74, 75]
Colon	-	+	-	[76]
Colorectal	-	+	+	[77, 78]
	-	+	-	[79]
Endometrial	-	-	+	[80]
Esophageal	-	+	+	[81, 82]
Neuroblastoma	-	+	-	[83]
Osteosarcoma	+	+	+	[84]
	-	-	+	[85]
Prostate	-	+	+	[86, 87]
Renal	+	+	+	[88, 89]

**Note:** +: Progress

## LIST OF ABBREVIATIONS

ASOs: Antisense Oligonucleotides
BC: Breast Cancer
EC: Esophageal Cancer
EMT: Epithelial-mesenchymal Transition
ESCC: Esophageal Squamous Cell Carcinoma
GC: Gastric Cancer
KSHV: Kaposi's Sarcoma-associated Herpesvirus
lncRNAs: long non-coding RNAs
MALAT1: Metastasis-Associated Lung Adenocarcinoma Transcript 1
MEN: Multiple Endocrine Neoplasia
MI: Myocardial Infarction
miRNAs: microRNAs
NSCLC: Non-small Cell Lung Cancer
QoL: Quality of Life
